# Application of a Sanger-Based External Quality Assurance Strategy for the Transition of HIV-1 Drug Resistance Assays to Next Generation Sequencing

**DOI:** 10.3390/v12121456

**Published:** 2020-12-17

**Authors:** Cheryl Jennings, Neil T. Parkin, Daniel J. Zaccaro, Rupert Capina, Paul Sandstrom, Hezhao Ji, Donald J. Brambilla, James W. Bremer

**Affiliations:** 1 Department of Molecular Pathogens and Immunity, Rush University Medical Center, Chicago, IL 60612, USA; james_bremer@rush.edu; 2 Data First Consulting, Inc., Sebastopol, CA 95472-2129, USA; nparkin34@gmail.com; 3 Research Triangle Institute (RTI) International, Research Triangle Park, NC 27709-2194, USA; dzaccaro@rti.org; 4 National HIV and Retrovirology Laboratories at JC Wilt Infectious Diseases Research Centre, Public Health Agency of Canada, Winnipeg, MB R3E 3L5, Canada; rupert.capina@canada.ca (R.C.); paul.sandstrom@canada.ca (P.S.); hezhao.ji@canada.ca (H.J.); 5 Department of Medical Microbiology and Infectious Diseases, Rady Faculty of Health Sciences, University of Manitoba, Winnipeg, MB R3E 0J9, Canada; 6 Research Triangle Institute (RTI) International, Rockville, MD 20852, USA; dbrambilla@rti.org

**Keywords:** HIV-1 drug resistance, next-generation sequencing, external quality assurance, Sanger sequencing

## Abstract

The National Institute of Allergy and Infectious Diseases (NIAID) Virology Quality Assurance (VQA) established a robust proficiency testing program for Sanger sequencing (SS)-based HIV-1 drug resistance (HIVDR) testing in 2001. While many of the lessons learned during the development of such programs may also apply to next generation sequencing (NGS)-based HIVDR assays, challenges remain for the ongoing evaluation of NGS-based testing. These challenges include a proper assessment of assay accuracy and the reproducibility of low abundance variant detection, intra- and inter-assay performance comparisons among laboratories using lab-defined tests, and different data analysis pipelines designed for NGS. In collaboration with the World Health Organization (WHO) Global HIVDR Laboratory Network and the Public Health Agency of Canada, the Rush VQA program distributed archived proficiency testing panels to ten laboratories to evaluate internally developed NGS assays. Consensus FASTA files were submitted using 5%, 10%, and 20% variant detection thresholds, and scored based on the same criteria used for SS. This small study showed that the SS External Quality Assurance (EQA) approach can be used as a transitional strategy for using NGS to generate SS-like data and for ongoing performance while using NGS data from the same quality control materials to further evaluate NGS assay performance.

## 1. Introduction

The National Institute of Allergy and Infectious Diseases (NIAID) Virology Quality Assurance (VQA) program was operated by Rush University Medical Center from 1993–2019 (Rush VQA). The main objective of this program was to provide a comprehensive quality assurance program for laboratories supporting NIAID-sponsored clinical trials. The Rush VQA collaborated with other funding agencies, including the Centers for Diseases Control and Prevention (CDC), the World Health Organization (WHO), NIAID-sponsored clinical trial networks, and individual client investigators. Its activities involved evaluating new virology assays [1,2,3,4,5], determining the impact of specimen type [6,7,8] and analyte stability on assay performance [9,10,11,12,13], and producing quality control materials for use as run controls, assay validation, or quality assessment [14,15,16,17]. In 2001, the Rush VQA rolled out an external quality assurance (EQA) program for Sanger sequencing (SS)-based HIV-1 drug resistance (HIVDR) genotyping. Early evaluations used dry panels to evaluate the impact of editing on the overall quality of the data [18]. While the participating laboratories clearly understood the guidelines for editing, most laboratories adopted internal guidelines and though this did not appear to be detrimental to the overall concordance of the data when using a commercial kit where training is provided, lack of standardized guidelines led to inconsistent editing practices. Later panels included coded plasma specimens [19] which were tested using local protocols and the data were submitted for analysis. This testing helped to set the groundwork for the future analysis of laboratory performance for SS HIVDR testing, which included the receipt and handling of specimens (pre-analytical phase), the extraction, amplification and sequencing of the panel specimens (analytical phase) and the reporting of amino acid substitutions associated with HIVDR (post-analytical phase). In the early years of testing, the majority of the laboratories that participated in the VQA HIVDR EQA program used research use only versions of manufactured kits that were eventually approved by the U.S. Food and Drug Administration (Applied Biosystems ViroSeq™ HIV-1 genotyping system (VS) and the Visible Genetics TruGene™ HIV-1 Genotyping kit (TG)) [19,20,21,22]. A few laboratories submitted data using laboratory-developed tests (LDTs). The Rush VQA sent out two panels per year (1–37 g) and included specimens with HIV-1 RNA concentrations ranging from 2000–100,000 copies/mL and HIV-1 subtypes A, CRF02_AG, AE, B, C, D, F, and G. At the onset of the program, 32–36 laboratories submitted data for panels 1–5 g, and these mainly comprised laboratories in the United States that participated in NIH-sponsored studies. Data collected from the first five panel distributions consisted of 14–18 VS data sets, 14–20 TG data sets, and 4–6 LDTS data sets. By 2018, the list of participating laboratories and assays used had changed dramatically to include 51 laboratories from 21 countries and 64 data sets: two NGS, 40 LDTs, and 22 VS.

Several other EQA programs have been described with similar approaches to monitoring HIVDR test quality [23,24,25,26,27,28]. Many similarities exist in the methods used for evaluating HIVDR testing; in fact, several laboratories from other programs also participated in the Rush VQA program [25,26,28]. Some programs used artificial specimens [25,27], while others, like the Rush VQA, opted to use clinical specimens for their evaluations [23,24,26,28]. While artificial specimens are useful for validating assay performance within a laboratory, they do not offer the same challenges as clinical specimens, which contain more heterogeneous virus populations with mixtures at individual nucleotide positions [19,23].

The main objective of an EQA program is to provide an unbiased approach for evaluating tests and laboratory performance. Since the authentic reference sequence of HIV in clinical specimens is unknown, one way to define the consensus is to combine data generated by participating laboratories to define a group consensus sequence against which each test sequence is compared. The Rush VQA used a minimum of seven data sets to generate a consensus for each panel specimen; less than 80% agreement at any position was sufficient to result in non-consensus. If insufficient data were available to define a kit-specific consensus, all available data were combined to create the consensus. The VQA used specific “watch regions” to evaluate sequence data: amino acids 4–99 for protease (PR), 38–247 for reverse transcriptase (RT), and 50–200 for integrase, covering all HIV drug resistance-associated mutations (DRMs) according to the International Antiviral Society–USA [29]. The consensus was evaluated at each nucleotide position within the watch region. If less than 80% agreement was noted at a nucleotide locus, an “n” was inserted, and that position would not be taken into account for the scoring. For instance, if 12 laboratories reported three As, three Gs and six Rs for a given nucleotide position, there was non-consensus for that position. In this case, the data were ignored for proficiency testing scoring. However, new technologies, such as next generation sequencing (NGS), offer a more quantitative way to evaluate HIV drug resistance, but the sensitivity of these assays and the clinical relevance of low abundant variants (LAVs) is not yet defined. In 2017, the Rush VQA, in collaboration with the WHO Global HIV-1 Drug Resistance laboratory network [30] and the Public Health Agency of Canada [31], invited laboratories that had developed HIVDR testing using NGS to participate in a pilot study, aiming to explore the feasibility of assessing NGS HIVDR data using Sanger sequencing (SS)-based EQA scoring strategies as a transitional approach for switching to NGS technologies while simultaneously collecting data for more in-depth analyses of NGS data and laboratory performance.

## 2. What Have We Learned from Sanger-Based EQA?

There are several lessons learned from the establishment of an EQA program for HIVDR testing. The purpose of an EQA program is to ensure the quality of testing, which may include the distribution of well-characterized quality control material to verify the performance specifications of a new assay, to verify the run performance or new reagent or kit lots of an existing assay, or to verify ongoing performance through proficiency testing. The quality control material (QCMs) used for each of these purposes may consist of the same type of material (e.g., the same human-derived material used for routine testing) or it may consist of contrived specimens, such as infectious molecular clones or cultured virus stocks that contain virus with specific HIV DRMs, and can be used to create specimens with “known” resistance patterns. Contrived specimens are likely to be more useful when a laboratory is verifying the performance specifications of an assay (i.e., assay validation), but are less useful in proficiency testing because they do not truly represent the specimens used for routine testing, and do not offer the same complexity and challenges associated with human-derived material containing many more diverse populations of viruses within the specimen. The challenge with using human-derived QCMs is not knowing what DRMs they contain and the exact abundance of each variant within the specimen. However, having access to well-characterized QCMs and a large laboratory network can help to generate data quickly for evaluating new assay performance. Whereas many new assays are commercially available with pre-defined assay performance specifications, some new assays are rolled out as laboratory defined tests (LDTs) where the assay performance is defined within a laboratory, but limited data exist for performance across assays and laboratories; such is the case with HIVDR testing with NGS. Assay validation within a laboratory is not only expensive, but it is less robust and the defined performance specifications may not translate to a second laboratory and the data may not correlate with data generated by another laboratory using a different LDT to perform the same kind of test. An EQA program can be used to collect and compare data generated across laboratories using the same technologies but different LDTs to see how the performance compares when using the same QCMs. While the Sanger-based detection of HIV DRMs was based on a pre-established cutoff defined by the early manufacturers of those kits, no pre-defined detection thresholds are defined for NGS. Zhou and Swanstrom suggest that using a 1% threshold for NGS is unrealistic due to the inherent frequency of errors generated during PCR, especially in assays that do not control for sampling depth within their assays [32]. Becker et al. claim that drug resistance can be reported with an accuracy from 2–100% based on testing done in two laboratories [33]. More data are needed to determine how performance varies the assay/pipeline, laboratory, and specimen, so while early analyses will help to define performance specifications, ongoing surveillance will be needed to verify that performance does not change over time. The larger the number of laboratories participating in the early phases of testing, the more robust the interpretation of the data.

It is critical to have standardized data collection so that raw data files may be imported into statistical programs without requiring manual manipulation prior to statistical analysis. Likewise, if HIV DRM reports are to be included in the scoring algorithm, any filters employed in generating data reports must be pre-defined to eliminate errors associated with internal filters used for local testing. Since it is unlikely that a single assay or pipeline will be utilized for NGS HIVDR testing, pre-defined raw data files must be submitted for analysis. The raw data files should be generated using a standardized threshold, e.g., a 1% cutoff, to allow statistical analyses to evaluate performance across assays in detecting LAVs. This will require the replication of specimens within runs, across runs, and across laboratories. Since viral load will impact the detection of LAVs, it will be important to define the lowest viral load that should be used for testing and define performance based on that viral load. Evaluations should also include specimens with higher viral loads to determine how higher viral loads impact HIVDR mutation detection, but ongoing proficiency testing should include specimens with a range of viral loads that would be expected in routine testing. If more than one type of specimen is used for testing, e.g., plasma vs. dried blood spots, then each performance specifications for each specimen type must be evaluated separately.

## 3. Can We Use Existing SS EQA Programs to Evaluate NGS Data?

It will take time before all laboratories transition to NGS technologies, and the existence of LDTs far outnumbers the existence of FDA-cleared tests with only one system [34] being FDA-cleared for HIVDR testing. This will clearly have an impact on ongoing clinical trials and HIV surveillance programs, as it will take time to gather sufficient data to permit changes in how data can or should be interpreted, especially with respect to the detection of LAVs. In this investigation, we applied the scoring criteria used for assessing SS HIVDR testing. The first goal was to show that NGS data can be used to generate Sanger-like data and can be assessed for evaluating NGS assay performance using the data generated from the same proficiency testing specimens. The idea is not to use a consensus FASTA file to evaluate NGS assay performance, but to generate equivalent data for ongoing laboratory performance using existing metrics until new metrics can be defined. Herein, we describe a mock quality assessment of NGS consensus sequences generated with a range of detection thresholds (5%, 10%, and 20%) to see how the data compare to Sanger-based consensus sequences generated for proficiency testing and to identify new challenges with NGS-based testing. Archived panels from the Rush VQA PT panels 24 g and 26 g, both containing five specimens, were used for this evaluation. The panels and the laboratories included in this evaluation have been previously described [35]. These panels were selected because they included specimens with considerable heterogeneity in drug resistance patterns and many non-consensus nucleotide positions in the consensus sequence derived during proficiency testing. The panels included specimens with HIV-1 B, C, D and F subtypes with a range of viral loads from 3656–29,139 copies/mL. All participating laboratories performed locally developed LDTs using Illumina MiSeq. The NGS data analysis pipelines used included HyDRA, PASeq, MiCall, Hivmmer or other internally developed bioinformatics tools [3,30,31]. The data collected from the laboratories for EQA assessment were the NGS consensus sequences (in FASTA format) for the PR and RT gene regions using mixed-base calling thresholds of 5%, 10%, and 20%.

**Figure 1:**
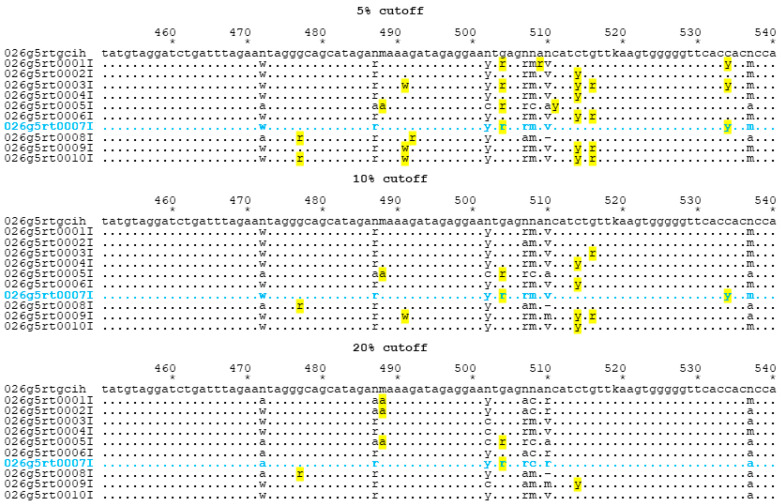
Alignment segment (451–540, RT amino acids 188–217) taken from RT sequences from panel 26 g, specimen 5 to illustrate stage 1 error details. Yellow highlighted bases differ from Sanger sequencing (SS) consensus. An “n” in the consensus (first) row indicates that no consensus was achieved in the original analysis. The blue highlighted row indicates laboratory 7, for which details about next generation sequencing (NGS) quantitative data are presented in Table 2.

All the NGS consensus FASTA sequences generated using the given thresholds were compared against the consensus sequences previously established using SS proficiency testing. The consensus used for this mock assessment was derived during proficiency testing by combining data from 30 participating laboratories who submitted data using a commercially-available kit (TruGene or ViroSeq) and determining consensus at each nucleotide position based on an 80% cutoff. The letter “n” in a consensus sequence indicates non-consensus at that position. Ultimately, consensus sequences should not be used to evaluate NGS data; however, this is the method used to evaluate SS and, therefore, it is the starting point for this evaluation. To evaluate the feasibility of using SS EQA methodology for evaluating NGS consensus sequences, we performed mock EQA assessments on these data using previously defined scoring criteria for SS assays [36]. Briefly, the consensus FASTA files were scored using a truncated scoring system (Stages 1 and 2 scoring only). Stage 3 scoring was not included in this mock analysis due to inconsistencies in NGS reporting. This highlights the need for standardized data reports for evaluating HIV DRM calls for proficiency, and the HIV DRMs used for scoring should be based on some pre-established criteria. Similarly, missing data (e.g., consensus FASTA files that did not span the entire watch region or had missing data in the middle of a gene region) were ignored (i.e., missing data were not scored as an error). This highlights the need to define whether or not data for entire gene regions need to be submitted or if scoring can be truncated if data are missing. Table 1 illustrates the details of scoring for RT for panel 26 g specimen 5, across cutoffs for mixed base determination. As expected, there was a higher error rate for the 5% cutoff as more mixed bases were detected, resulting in more discrepancies with the SS consensus base calls. Most laboratories produced enough errors at the 5% cutoff to yield a *p*-value < 0.01, and consequently received more penalty points than at the 20% cutoff. This highlights the limitations of SS data. Nucleotide base calls and mixture calls are based on the threshold used for generating a consensus sequence and nucleotide mixtures that exist in frequencies near the threshold limit will result in variability at that locus. Details of a representative portion of the sequence alignment for VQA panel 26 specimen 5 RT using the three cutoff values (5%, 10%, 20%) are shown in Figure 1. Segments of the alignments constructed for this specimen for all three cutoffs are annotated to illustrate the effect of different cutoffs on nucleotide base calls vs. mixture calls, which translates to increased error rates. Sequences for laboratory 7 are highlighted in blue for comparison with quantitative NGS nucleotide detection frequency rates presented in Table 2. Total scores for each panel for NGS consensus sequences at all thresholds are shown in Table 3. Some of the NGS sequences did not cover the entire VQA watch region; missing data were not included in the calculations of homology or in the error counts in order to focus on true differences in the data. Scores for errors in PR and RT gene regions for all specimens in each panel were combined to create a panel score using 5%, 10%, and 20% thresholds. For 24 g, 6 out of 10 laboratories received provisionally certified scores for data generated with a 5% threshold (median score = 9), but none received provisional scores for data generated using a 10% threshold (median score = 2) or 20% threshold (median score = 1). For 26 g, 7 out of 10 laboratories received scores of PC for data generated with a 5% threshold (median score = 9), compared to two laboratories that received scores of PC for data submitted with a 10% threshold (median score = 3) and one for data generated with a 20% threshold (median score = 2). Three laboratories (3, 7, and 8) received scores of “certified” (C) for all analyses.

While consensus FASTA files can be used to generate SS-like data for ongoing performance, nucleotide frequency data generated using a 1% threshold should also be generated for the ongoing analysis of NGS data. For equivalence testing and proficiency, labs should submit NGS-generated consensus FASTA files created using the 20% threshold, but for NGS evaluations, they should submit a standardized nucleotide frequency detection report for more in-depth analysis of NGS data.

## 4. Discussion

Before an EQA program for NGS-based HIVDR assays can be established, the performance expectations must be defined. If the assay performance is not yet determined, data must be generated to help set expectations. Using EQA panels is one way to generate data for determining assay performance. Having a fundamental understanding of how a HIVDR EQA program works for SS assays is essential when thinking about moving forward with evaluating NGS-based sequencing data in the future.

While some EQA programs use infectious molecular clones to evaluate HIVDR performance, the viruses in these specimens are more homogeneous than those in clinical specimens, usually containing highly diversified viral populations [19,23]. Clinical specimens are better for monitoring laboratory performance, but also present challenges associated with defining the “ground truth” of each sample, including the authentic consensus, the DRMs they contain, and the exact abundance of each variant.

One of the lessons learned from EQA programs for SS is that large amounts of data can be generated during proficiency testing and provide a basis for inter-assay and inter-laboratory performance comparisons. The use of standardized electronic reporting for the collection of EQA data helps to facilitate statistical analyses and minimize errors associated with manual transcription. Replicate specimens within and between panels that include a range of viral loads can help to generate temporal trend data for evaluations of sensitivity, precision, and accuracy of mixture reporting. However, viral loads used for PT should not challenge the sensitivity of the assay to the point where false negatives can occur by chance, but they should also not be so high that they mask minor problems.

Since EQA programs for SS HIVDR currently exist, a logical approach to EQA for NGS is to use the existing programs to monitor NGS laboratory performance and use the same specimens to further evaluate NGS assay performance. Using this approach of submitting one form of data for ongoing proficiency while submitting alternate data files, such as standardized nucleotide sequence variant frequency reports, will permit the simultaneous accumulation of NGS data from multiple laboratories using standardized specimens for investigational purposes without incurring additional costs for testing. The EQA program can adjust proficiency testing panel configurations to address specific questions such as reproducibility within and across panels, and the effect of viral load on low abundance variant detection. The obvious first step is to see how NGS-derived data compare to the existing SS. In the current study, we used NGS-generated Sanger-like FASTA files, created using 5%, 10%, and 20% thresholds, to compare with SS data. The first step in evaluating NGS data is to treat the output like SS and score those files using well-established criteria such as those used by the Rush VQA. Based on VQA scoring, NGS consensus FASTA files created with thresholds of 20% yielded the best scores when compared to Sanger-based data, but more data are needed to determine if lower thresholds can yield comparable data. Three laboratories received passing scores for all panels and thresholds when compared to SS data, suggesting that some NGS assays may be better optimized and could offer more robust data even at lower thresholds. These results are consistent with a parallel investigation of optimal variant thresholds for comparability between SS and NGS [35].

Quantitative NGS nucleotide frequency data containing the type of information in Table 2 provide us with the opportunity to extract more information to answer questions related to NGS, whereas with SS data, we were confined to nominal data methods (match, partial mismatch, complete mismatch). With quantitative results, we would be able to entertain quantitative statistical methodologies to assess laboratory performance, including the following statistical approaches:

1. Quantitative measures of laboratory performance: for example, for a given sample, gene region and nucleotide position, the percent distribution for each nucleotide base may be evaluated, summary statistics computed, and outliers identified based on parametric (e.g., are the data outside a three standard deviation window?) or non-parametric (e.g., are the data below the 5th or above the 95th percentile compared to the distribution from all other laboratories?).
2. Multivariate statistical techniques may be considered to evaluate the multivariate distribution of percentages of all four nucleotides. For example, the distribution of percentages may be considered in a four-dimensional space with a mean vector and correlation (or covariance) matrix. From these parameter estimates, suitable quantities to evaluate each laboratory in four-dimensional space may be computed; for example, Mahalanobis’s Distance is a measure of the “statistical distance” of each laboratory’s data point to the mean four-dimensional vector, and would quantify how far that laboratory is from the four-dimensional average in multivariate distance, accounting for correlations among the four percentages within a laboratory + nucleotide position. A suitable criterion could be developed to quantify whether a laboratory is within or outside acceptable boundaries. If traditional assumptions (e.g., Gaussian distributions) are violated, suitable transformations (e.g., natural logarithm) or alternative modelling (e.g., zero-inflated distributions) may be necessary.
3. Beyond evaluation of individual laboratories, an assessment may be made of how to quantify a point estimate of what the most likely base call should be (e.g., what is the most likely nucleotide at a given location?) and the confidence in that estimate (e.g., a confidence interval using the estimated covariance matrix). Quantitative criteria would also allow for “no consensus” results, i.e., there is no clarity on what nucleotide is the best call.

The standardization of file outputs will be critical for electronic capture of the data for statistical analysis without adding extra burden on the laboratory or adding to data management problems that arise from the manual transcription of data.

## 5. Conclusions

These data suggest that the SS-based EQA strategy may serve as a transitional solution for evaluating the performance of a laboratory conducting NGS-based HIVDR assays. The NGS consensus sequences created using thresholds ranging from 10% to 20% yield comparable scoring/ranking outputs with SS. However, additional studies are needed to better characterize a NGS HIVDR test and fully define the performance expectations for such assays, especially concerning sensitivity and the quantitative detection of low abundance DRMs. Once those performance expectations are defined, the new criteria can then be applied to proficiency testing scoring for the ongoing monitoring of laboratory performance for HIVDR using NGS.

## Figures and Tables

**Table 1:** Stage 1 errors (total errors, partial mismatches, complete mismatches) in reverse transcriptase (RT) for a representative specimen (5) from Virology Quality Assurance (VQA) panel 26 g.

Laboratory	5% Cutoff			10% Cutoff			20% Cutoff		
	% Homology *	Stage 1 Errors	Poisson *p*-Value	% Homology *	Stage 1 Errors	Poisson *p*-Value	% Homology *	Stage 1 Errors	Poisson *p*-Value
1	99.0	5		99.6	2		99.4	3	
2	98.3	10	0.021	99.1	5		99.1	5	
3	97.6	14	0.0004	99.0	6		100	0	
4	98.1	11	0.009	99.3	4		99.8	1	
5	97.2	16	<0.0001	97.2	16	<0.0001	97.4	15	0.00011
6	97.9	12	0.003	99.3	4		100	0	
7	98.3	10	0.021	99.1	5		99.5	3	
8	98.1	11	0.009	98.3	10	0.021	98.3	10	0.02138
9	98.1	11	0.009	98.1	11	0.009	98.3	10	0.02138
10	97.4	15	0.0001	99.1	6		100	0	

* % Homology was calculated by dividing the number of bases submitted that matched the group consensus sequence by the number of bases in the group consensus sequence after excluding non-consensus positions (Ns). Missing bases (89) for laboratory 1 are not included in the homology calculation and are not considered errors for Stage 1 (Poisson) error point calculations.

**Table 2:** Quantitative data from laboratory 7 for NGS for the same alignment data presented in Figure 1. Data are provided only for positions where the detection was less than 99% for a single base at a nucleotide position; detection rates < 1% are left blank. Position = locus numbering is the same as that used in the alignments used for VQA scoring; depth = number of reads at a given nucleotide position; A, C, G, T = base call detection percentages from NGS data.

Position	Depth	A	C	G	T
472	23,583	87			13
487	19,894	75		24	
488	19,907	76	24		
502	21,915		78		22
504	20,530	29		71	
507	21,464	59		41	
508	21,617	16	84		
510	20,543	57	17	25	
519	26,631			61	39
537	27,318	86	13		

**Table 3:** Mock EQA scoring outputs for NGS consensus sequences using different thresholds.

Lab	24 g Total Points, Score						26 g Total Points, Score					
	5%		10%		20%		5%		10%		20%	
	Points	Score	Points	Score	Points	Score	Points	Score	Points	Score	Points	Score
1	13	PC	4	C	2	C	9	PC	1	C	2	C
2	8	PC	0	C	0	C	14	PC	2	C	2	C
3	6	C	2	C	0	C	6	C	3	C	0	C
4	9	PC	2	C	5	C	8	PC	0	C	0	C
5	9	PC	6	C	3	C	9	PC	9	PC	8	PC
6	6	C	2	C	0	C	12	PC	6	C	3	C
7	3	C	2	C	0	C	3	C	2	C	0	C
8	3	C	3	C	4	C	4	C	3	C	3	C
9	9	PC	5	C	1	C	12	PC	8	PC	7	C
10	10	PC	1	C	0	C	9	PC	1	C	0	C

Total errors (stage 1 and 2) for all fives specimens for protease (PR) and RT combined within the panel are tallied by the laboratory, gene, % thresholds for NGS consensus generation and external quality assurance (EQA) scoring of the laboratory performance when NGS consensus at various thresholds was applied. Missing data were not included in the error counts because some data sets did not include sequences for the entire examined region. PC = provisionally certified (shaded cells; scores of 8–14; problems noted) and C = certified (scores of 0–7; no major problems noted).
